# Supplementary material for: Matrix-based imaging through dynamic scattering
Source: Nat Commun. 2025 Oct 24;16:9413. doi: 10.1038/s41467-025-64422-x (PMC12552628; doi:10.1038/s41467-025-64422-x)
Supplement: Supplementary file 1 — Supplementary information file [file 41467_2025_64422_MOESM1_ESM.pdf]

Matrix-based imaging through dynamic scattering - Supplementary Materials

Elad Sunray[1†], Gil Weinberg[1†], Benzy Laufer[1†], Ori Katz[1*]

[1]Institute of Applied Physics, The Hebrew University of Jerusalem, Jerusalem, 9190401, Israel.

*Corresponding author(s). E-mail(s): orik@mail.huji.ac.il;
†These authors contributed equally to this work.

1 Point spread functions estimation by frame-wise deconvolution

Since in isoplanatic imaging, each captured frame I_m is given by a convolution of the object, O, with the relevant PSF, P_m: $I_m = P_m * O$. Once I-CLASS retrieves the object, the PSF can be estimated by deconvolving each frame with the recovered object. This approach enables a study of the temporal dynamics of the medium from the estimated PSFs throughout the multi-frame video acquisition. To demonstrate this, we present two examples from the incoherent imaging experiments using Wiener deconvolution in Fig. S1 and supplementary videos S1 and S2, corresponding to the frames shown in Fig. 2b-e and 2f-i, respectively, in the main text. Fig. S1a,d shows a few raw captured frames, Fig. S1b,e show the reconstructed objects, and Fig. S1c,f show the estimated PSFs for each camera frame obtained using a Wiener deconvolution.

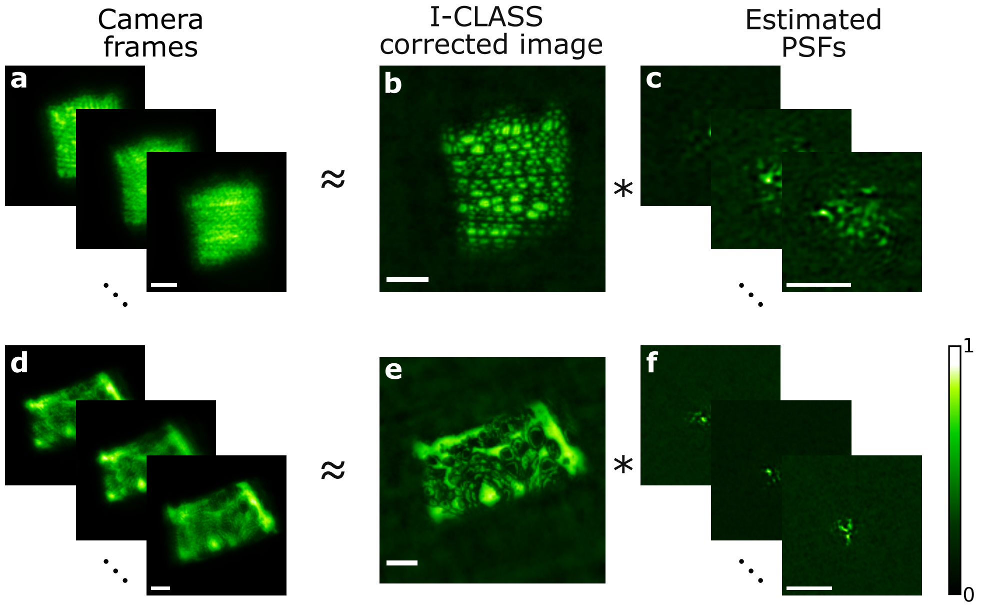

Fig. S1 Frame-wise estimation of time-varying PSFs using Wiener deconvolution. **a,d** shows the initial distorted frames corresponding to the frames presented in Figure 2(b,f) in the main text. **b,e** displays the I-CLASS retrieved object used to estimate the distorted object in frames d and h in Figure 2. **c,f** shows the PSFs obtained by deconvolving this estimated object from the initial distorted frames. This demonstrates the method's effectiveness in capturing frame-by-frame PSF variations over time. A video showing all retrieved PSFs using frame-wise deconvolution is given in supplementary videos S1-2. Scale bars, 100 µm.

To validate the uncorrelation condition of the PSFs required for I-CLASS and the reflection matrix estimation from the covariance matrix, we calculated the covariance matrices for the estimated PSFs both across camera frames (Fig. S2b) and across camera pixels (Fig. S2c). Due to the memory limitations imposed by the size of the pixel-wise covariance matrix, we restricted our calculation to the middle column of the

image (Fig. S2c). The results, displayed in Fig. S2, illustrate these covariance matrices corresponding to the PSFs presented in Fig. S1a–c. The frame-wise correlations presented in Fig. S2b are given after re-normalizing all estimated PSFs to have equal energy.

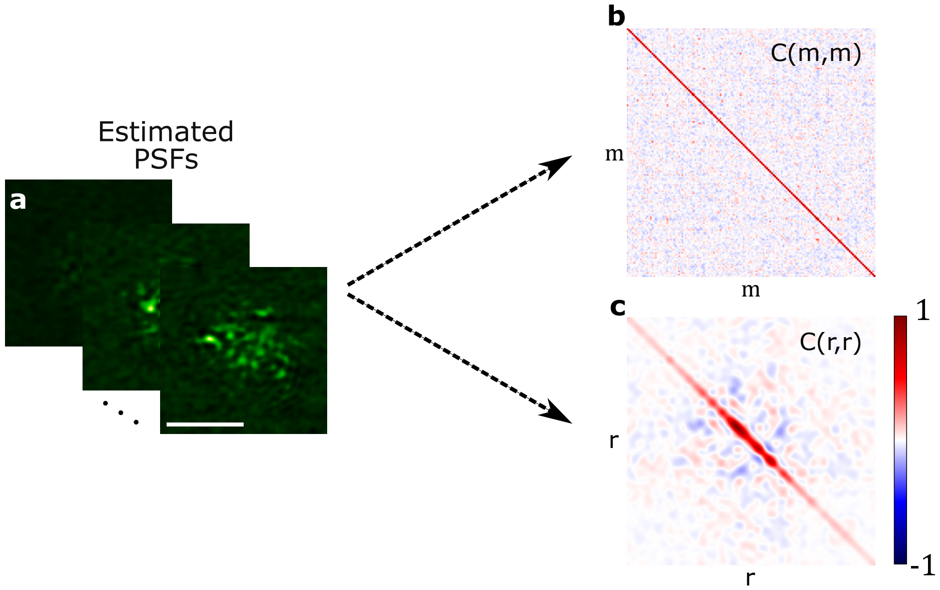

Fig. S2 Verifying the uncorrelation condition of P-matrix. **a** the initial estimated PSFs, similar to Fig. S1c, obtained by deconvolving the retrieved object with the captured distorted frames. **b** displays the frame-wise correlation between different PSFs, and **c** illustrates the pixel-wise correlation between the estimated PSFs, calculated for the middle camera row due to memory limitations. Here, $M = 150$ and $N = 500^2$. Scale bars, 100 µm.

Additionally, we provide supplementary videos S1 and S2 that show each frame of I_m alongside its estimated PSFs. These videos underscore the temporal dynamics in PSF variations and the utility of frame-wise deconvolution in PSF reconstruction.

2 Energy conservation as a source of correlation to the PSFs

A key assumption in our approach is that the PSFs matrix, $\mathbf{P}$, which contains the different time-varying PSFs at its columns, consists of uncorrelated speckle intensity patterns, with a spatial correlation determined by the detection speckle grain size δx. This assumption results in the covariance matrix of $\mathbf{P}$, being a predominantly diagonal matrix, with off-diagonal correlations extending to δx outside the diagonal (Fig. S3a). This diagonal's effective 'width' is dictated by the detection PSF, governed by the illumination numerical aperture (NA) and detection wavelength λ. However, when the PSFs originate from phase-only distortions, they have the same total energy, which leads to residual correlations between the different speckles that compose the PSF: e.g., if one speckle grain is brighter and has more energy, the remaining speckle grains in the PSF must have less energy, and vice versa. The effect is naturally more substantial, and the smaller the number of speckles contained in the PSF.

This fundamental effect introduces off-diagonal spatial correlations in the matrix $\mathbf{P}$ (Fig. S3b), which tend to reduce the quality of the reconstruction, as expected due to the covariance matrix being not diagonal as required for CTR-CLASS [1] and I-CLASS [2]. This is demonstrated in Figure S3c, where the experimental frames of Fig.2b-e, captured through a phase-only diffuser, were processed directly by the I-CLASS algorithm. The reconstructed image (Fig. S3c) shows the main features of the object on top of a varying hazy, slowly varying background. Post-processing can suppress this effect by multiplying each camera frame by a different fixed scalar factor. Thus, PSFs of different total energy are artificially generated. Fig. S3d shows the reconstruction using the same data as Fig. S3c but by applying I-CLASS after the scalar multiplication of the captured frames, with multiplication factors linearly varying from 1 to 2 with the frame numbers 1-150. Importantly, as expected, this phenomenon only occurs in our experiments that induce stepper motor rotation of a small-angle scattering element, such as the 0.5° holographic diffuser in Fig. 2b-m and Fig. 4 of the main text, and not in the experiments that utilize the 1° holographic diffuser (Fig. 2n-q), due to the relatively small number of speckles in the PSF in these cases.

To visualize the source of the effect of energy conservation on the covariance matrix, we present the covariance matrix of a numerically simulated dynamic scattering matrix $\mathbf{P}$, with and without energy conservation (Fig. S3a and b, respectively). The impact of energy conservation is evident in the non-negligible (negative) correlations observed in the off-diagonal elements. This is further illustrated by displaying a cross-section of the covariance matrix, showing the negative correlations around the point of interest due to energy conservation.

As discussed above, we addressed the off-diagonal correlations in the covariance matrix that are introduced by energy conservation by applying variable intensity modulation (scaling) across the captured frames. This was done by simply multiplying each captured frame by a scalar factor. We empirically found that modulating by factors between 1 and 2 gave near-optimal results. In Fig. S4 we present the reconstruction fidelity as a function of the choice of modulation depth for the experimental results of Fig. 2 in the main manuscript. To generate this result, we have performed multiple

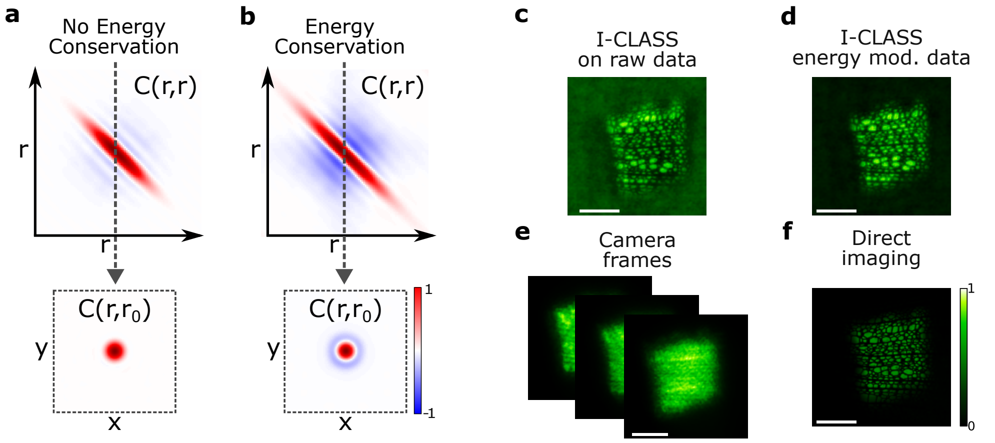

Fig. S3 Experimental reconstructions under energy conservation. a The covariance matrix of numerically dynamic scattering matrix **P**, displayed with and without energy conservation (**b**). The effect of energy conservation is highlighted by the negative correlation observed in the off-diagonal elements, further illustrated through a cross-section of the covariance matrix. The positive correlation in both cases indicates the influence of finite speckle size. **c,d** Experimental comparison of I-CLASS reconstructions with and without the application of intensity modulation for energy conservation. The reconstruction with energy modulation shows a reduction of intensity haze outside the reconstructed target. **e,f** Several camera frames and images taken without the scattering are provided for reference, using the optical setup in Fig. 2 of the main text.

reconstructions on the same experimental dataset, where before each reconstruction, a different value of varying amplitude modulation (scaling) was applied. For each depth value, we applied a linear scaling to our experimental data where the multiplication factor for the m-th frame (out of M total frames) was:

$$f_m = 1 + \frac{m-1}{M-1} \cdot (\alpha - 1) = 1, \ldots, \alpha \tag{1}$$

Where α controls the total range of modulation with corresponding modulation depth: $\frac{\max(f_m)-\min(f_m)}{\max(f_m)+\min(f_m)} = \frac{\alpha-1}{\alpha+1}$.

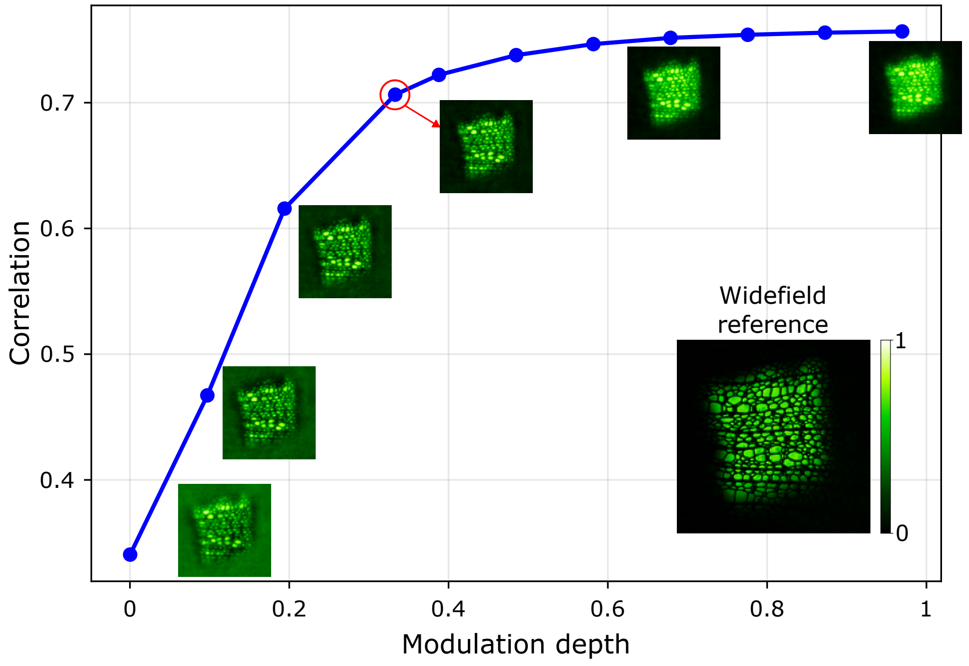

Fig. S4 Incoherent imaging reconstruction fidelity as a function of post-processing intensity modulation of captured frame. Analysis of the reconstruction fidelity for the experimental results of Fig.2d as different levels of post-processing intensity modulation are applied before running the I-CLASS reconstruction algorithm. The graph presents the Pearson correlation between the reconstruction and the widefield reference (bottom-right inset) as a function of the applied modulation depth (see text). Insets display sample reconstructions, showing the progressive suppression of background haze as the modulation depth increases. At higher modulation depths, the contrast within the object itself is lowered. The red circle indicates the modulation depth chosen for the incoherent reconstructions.

Our results show that without adding a modulation (modulation depth = 0), the reconstructions display a significant background haze. As the modulation depth increases, this background haze diminishes progressively. At very high modulation depths, while the background continues to reduce, the contrast is somewhat lowered, which may be the result of an imperfect estimation of the MTF in the I-CLASS algorithm [2]. Modulation depths of 0.3-0.5 used in our main experiments provide an effective trade-off between these competing effects. Additional improvements for addressing this point, such as SVD filtering, will be the focus of future work.

3 Comparison with speckle-correlation phase-retrieval based reconstruction

In this section, we present a numerical comparison between our proposed matrix-based approach for the incoherent imaging case and the established speckle correlation phase-retrieval based imaging [3, 4]. Inspired by Labeyrie's 1970 stellar speckle interferometry [5], speckle correlation imaging [3, 4]. is composed of two steps: the first is an estimation of the object autocorrelation (that is, its power spectrum) from the measured speckle patterns autocorrelation (or power spectrum); and the second is a subsequent reconstruction of the object from this estimated power-spectrum using a phase-retrieval algorithm [6]. Both methods utilize similar experimental setups and image acquisition schemes, allowing for a direct performance evaluation. To perform this comparison, we focused on several numerically simulated isoplanatic imaging scenarios. Our results demonstrate the superior performance of the matrix-based I-CLASS approach when reconstructing complex non-sparse natural target objects.

Importantly, in contrast to the I-CLASS reconstruction algorithm that has no free parameters except for the number of iterations, the estimation of the object's autocorrelation from measured speckle patterns and the phase retrieval algorithm require very careful optimization and tailoring of signal conditioning, including envelope estimation and correction, windowing and background subtraction, as well as fine-tuning of the phase-retrieval reconstruction algorithm parameters, including specific support constraints, number of iterations, choice of beta parameter, and number of independent runs with random initial guesses. The reconstruction fidelity obtained by phase retrieval of speckle correlations is extremely sensitive to the exact choice of signal conditioning and parameters, and without a precise and careful optimization of this rather large set of parameters, the reconstruction fidelity may be very poor for complex non-sparse objects.

Figure S5 presents the results of this numerical study. Each of the two rows of panels in Fig. S5 displays the results for a different target object. For each target object, we display the widefield reference image of the object taken without the scattering medium present (Fig. S5a,f), a sample simulated captured frame distorted by scattering (Fig. S5b,g), the speckle correlation phase-retrieval reconstruction (Fig. S5c,h), the matrix-based I-CLASS reconstruction (Fig. S5d,i), and the result of the same phase retrieval algorithm applied directly to the reference image power spectrum (Fig. S5e,j).

In all of the cases tested, our matrix-based approach demonstrated superior reconstruction fidelity than speckle correlations phase-retrieval based reconstructions. Most importantly, the I-CLASS matrix-based algorithmic reconstruction is extremely stable, providing solid results in every run, does not require any free parameters, signal conditioning, or other tweaks that speckle-correlation phase-retrieval requires for achieving a reasonable reconstruction. Strikingly, the matrix-based reconstruction demonstrates superior reconstruction quality even when compared to the best phase-retrieval speckle-correlation reconstruction obtained from 500 independent runs of the phase-retrieval algorithm on the optimized estimated power spectrum.

For producing Supplementary Fig. S5, we have applied the implementation of I-CLASS algorithm given in Weinberg et al.[2] directly on **a set of 100** simulated

captured frames. These frames were generated by convolving the original target image with 100 different random simulated speckle PSFs in the Fourier domain. For the speckle-correlation reconstruction, we employed the reconstruction approach of Bertolotti et al. and Katz et al. [3, 4] **using the same set of 100 simulated distorted images**, following a rigorous optimization of the choice of signal conditioning and algorithmic parameters, which are as follows:

1. Calculate the autocorrelation of each scattered light intensity frame.
2. Average all autocorrelations. The resulting autocorrelation suffers from an envelope given by the average envelope of the scattered light patterns.
3. Correct the autocorrelation envelope by dividing the result of step (2) by the autocorrelation of the mean of the images. This provides an estimate of the envelope of the PSF and normalizes this envelope from the raw average autocorrelation of all frames.
4. Subtract the minimum value of the resulting autocorrelation to account for the near constant background of 2:1 speckle intensity autocorrelation [4].
5. Apply appropriate windowing for optimal phase-retrieval results: a square-root of a Tukey window for the USAF target and a Tukey window taken to the power of 1/4 for the camera-man target.

For phase retrieval, following Katz et al. [4], we ran 500 independent runs of the following phase-retrieval algorithms, where in each run a different random initial guess for the object was used, and the lowest error reconstruction is presented in Fig. S5:

1. 300 iterations of HIO algorithm with a decreasing value of beta parameter, from 2.0 to 1.2, in steps of 0.2. A total of $5 \times 300 = 1,500$ iterations.
2. 300 additional iterations of error reduction algorithm.
3. In each iteration, we applied the following object-dependent constraints:
   - For the cameraman: real, positive, and non-negative constraints
   - For the USAF target: same constraints plus an additional finite square support constraint.

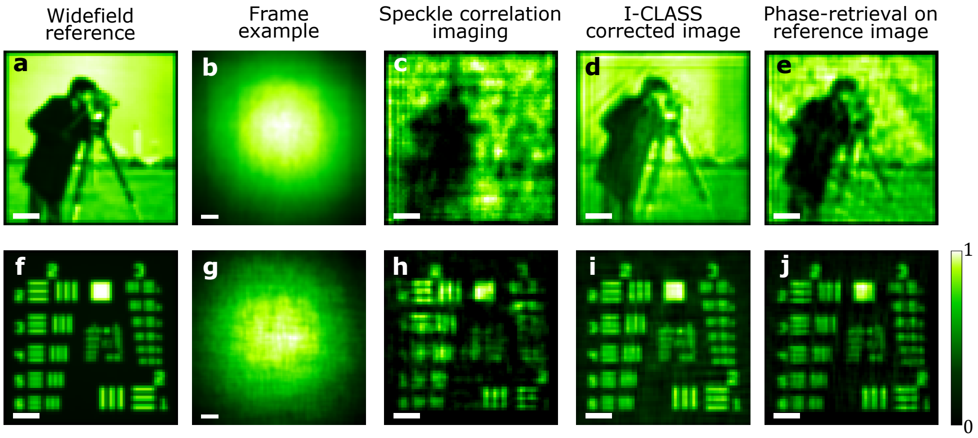

Fig. S5 Numerical comparison of imaging through dynamic scattering media with our matricial approach vs. speckle-correlation phase-retrieval based imaging. A comparison between the matrix-based I-CLASS reconstruction and speckle correlation phase-retrieval based imaging [3, 4]. **a,f** Simulated target objects. **b,g** Sample simulated captured single camera frames under dynamic scattering. **c,h** Best results of speckle correlation imaging reconstructions from 500 runs of HIO phase-retrieval algorithm, followed by error-reduction algorithm, after careful optimization of object autocorrelation estimation (see details in text). **d,i** I-CLASS reconstructions showing superior fidelity from every single run with no free parameters or signal conditioning. **e,j** Phase retrieval applied directly to the reference images power spectrum, for comparison. Scale bar: 10 px.

4 Digital autofocus by Fresnel propagation

In holographic imaging through scattering media, a key advantage is the ability to numerically propagate the reconstructed complex field to any desired plane once the field is retrieved at a single reference plane. This capability, often termed digital autofocus, eliminates the necessity of knowing the exact object distance during data acquisition or during the I-CLASS iterations.

In our coherent imaging experiments (Fig. 5), the I-CLASS algorithm reconstructs the complex-valued object field at the scattering layer plane ($z = 0$). This field can be digitally propagated to any desired plane using the Fresnel propagation operator (or angular spectrum propagator) described in the Methods section of the main text (**"Fresnel propagation via Fourier-domain transfer function"**).

By computationally varying the propagation distance and observing the resulting reconstructed intensity distributions, we identify the optimal object plane where the finest features of the target come into focus. This process is analogous to the physical process of adjusting the focus in a conventional microscope, but performed entirely in post-processing.

Figure S6 demonstrates this capability by showing the reconstructed field intensity at three distinct propagation distances: before the object plane ($z = 5.3\,\text{cm}$), at the object plane where optimal focus is achieved ($z = 7.15\,\text{cm}$), and after the object plane ($z = 9\,\text{cm}$). The sharp focus observed at $z = 7.15\,\text{cm}$ confirms that this is indeed the correct object plane, having the highest resolution of the fine features of the USAF target.

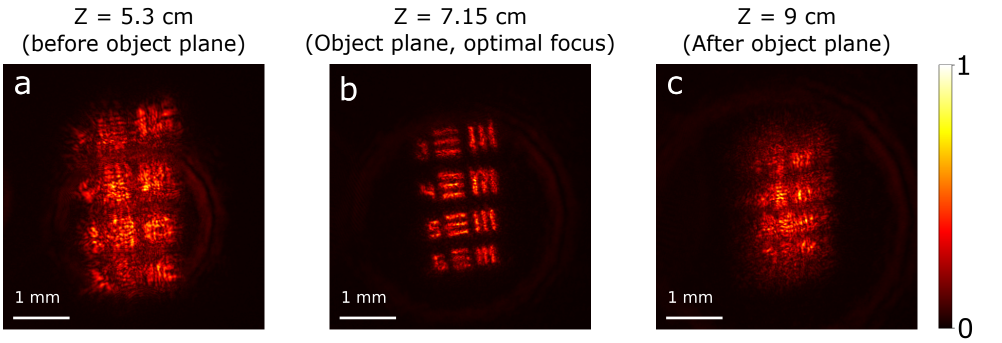

Fig. S6 Digital autofocus capability of the coherent imaging reconstruction. The I-CLASS algorithm provides the complex-valued object field at the scattering layer plane. This field can be back-propagated to any desired distance from the scattering layer, allowing to find the target object plane by 'digital focusing' in post-processing, without prior knowledge of the target position. Presented images show reconstructed field intensity at three propagation distances: **a** $z = 5.3\,\text{cm}$ (before the object plane), **b** $z = 7.15\,\text{cm}$ (at the object plane, where optimal focus is achieved), and **c** $z = 9.0\,\text{cm}$ (after the object plane). Scale bars: 1 mm.

5 Effect of illumination homogeneity and stability on coherent imaging through dynamic scattering

In our coherent imaging demonstration (Fig. 5 of the main text), maintaining a constant homogeneous illumination pattern at the object plane despite the dynamic scattering introduced by the rotating diffuser is important to ensure the 'fixed object' assumption of our model (Eqs. 6-7 of the main text). While obtaining a constant homogeneous illumination through a dynamic scatterer is rather straightforward with spatially-incoherent illumination, it is often challenging when spatially coherent illumination is considered. Nonetheless, it can be achieved for the case of a dynamic *thin* scatterer, such as a diffuser, by focusing the illumination spot size on the diffuser, such that the spot size is sufficiently smaller than the diffuser's coherence (or correlation) area. In this case, the illumination beam effectively experiences propagation through a single coherence area with a nearly constant phase function, and thus does not experience scattering, providing a homogeneous illumination of the target. Fig. S7 demonstrates and numerically studies the limitation of this approach. The top row (Fig. S7a-c) shows the phase pattern of a thin scattering layer with the illumination spot superimposed for three cases: where the illumination spot diameter is 0.1, 0.3, and 0.5 times the diffuser correlation length ($d_{\text{correlation}}$), respectively. The second row (Fig. S7d-f) shows the resulting illumination intensity pattern at the object plane after the light has propagated through the diffuser, simulated using angular spectrum propagation. The third row (Fig. S7g-i) shows the effect of these illumination patterns on the effective object (the product of the object and the illumination), while the fourth row (Fig. S7j-l) shows the reconstruction results.

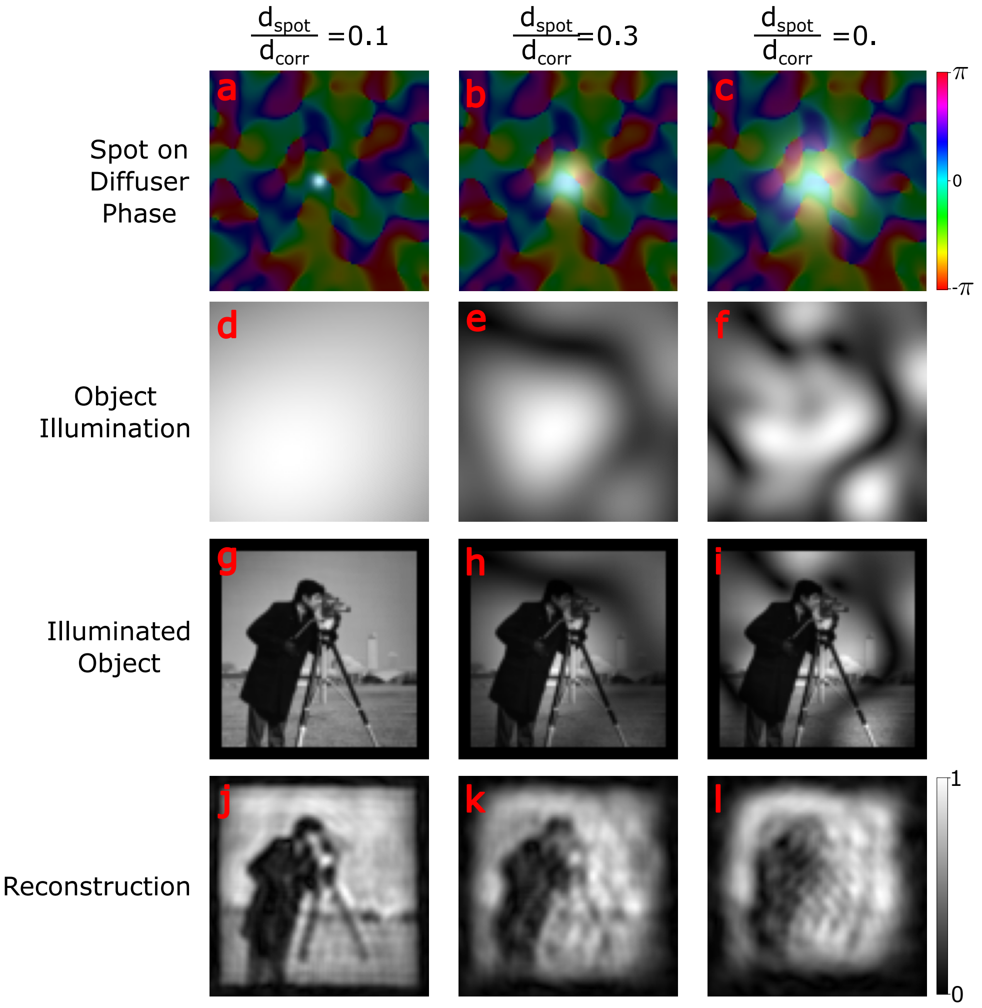

Fig. S7 Effect of illumination spot size on imaging through dynamic scattering. Numerical simulations demonstrating how the ratio between the illumination spot size (d_{spot}) to scattering layer (or diffuser) correlation length (d_{corr}, the square root of the scattering layer coherence area) affects the illumination at the object plane, and reconstruction quality. Each column represents a different spot size to correlation size ratio: $\frac{d_{\mathrm{spot}}}{d_{\mathrm{corr}}} = 0.1$ (left), $\frac{d_{\mathrm{spot}}}{d_{\mathrm{corr}}} = 0.3$ (middle), and $\frac{d_{\mathrm{spot}}}{d_{\mathrm{corr}}} = 0.5$ (right). **a-c** Scattering layer phase pattern (color) and illumination spot size at the scattering layer surface (bright highlighted area). The colorbar indicates phase values from $-\pi$ to π. **d-f** Resulting illumination intensity patterns at the object plane after numerical propagation through the scattering layer and free space. The grayscale colorbar indicates normalized intensity. **g-i** Effective object reflected field (i.e., that target object reflectivity profile multiplied by the illumination pattern). **j-l** I-CLASS reconstructed images from 150 scattering realizations. Note how the small illumination spot size relative to the scattering layer coherence area (left column) maintains a relatively uniform illumination at the object plane, and enables high-quality reconstruction, while larger spot sizes (middle and right columns) create varying speckle patterns, which degrade reconstruction quality.

When the illumination spot is much smaller than the scattering layer coherence area (Fig. S7a), it effectively experiences a nearly constant phase, providing a relatively uniform illumination at the object plane. This consistency across different realizations of the scattering layer is required to maintain the assumption of our imaging model. Indeed, the I-CLASS reconstruction under these conditions is of rather high quality. Note that there exists a global phase shift of the illumination, but this global phase shift can be mathematically contained in the detection PSF, keeping the effective field reflected from the object fixed.

In contrast, when the illumination spot size is comparable to or larger than the diffuser correlation length (Fig. S7c), the beam simultaneously samples multiple uncorrelated regions of the diffuser. This produces complex speckle patterns at the object plane that vary significantly between diffuser positions, creating a different illumination pattern for each diffuser realization. This variation violates our assumption of constant illumination in Eqs. 6-7 of the main text. As a result, the reconstruction quality gradually degrades as the spot size increases relative to the scattering layer coherence area.

In our experimental implementation described in the main text, we carefully focused the beam to ensure a spot size smaller than the diffuser's correlation length, thereby maintaining sufficiently constant spatial illumination patterns at the object plane across different realizations, as required.

6 Dependence of reconstruction fidelity on number of realizations, object sparsity, and SNR

To study the dependence of the reconstruction quality on the number of realizations, object complexity, and SNR, we carried an in-depth numerical study where we numerically simulate reconstructions using a different number of random realizations (M) for different object complexities (as given by the object sparsity) for both incoherent and coherent imaging scenarios. The results of these analyses are presented as reconstruction fidelity heatmaps in Fig. S8 To ensure statistical robustness, each data point in these heatmaps represents the average outcome from 10 independent numerical experiments.

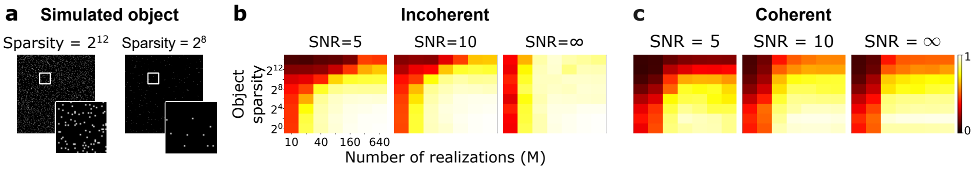

Fig. S8 Reconstruction fidelity as a function of the number of realizations, signal to noise (SNR), and object sparsity. Numerical results studying the reconstruction fidelity as a function of the imaging parameters. Simulated objects (a) consist of a varying number (sparsity) of bright points over a dark background. For each studied case, defined by object sparsity, SNR, and number of realizations, 10 distinct numerical experiments with different random distributions and scattering realizations were simulated and reconstructed. For all simulations, a camera pixel count of $N = 350 \times 350$ was used, and an $\sim$18-pixel wide scattering PSF. Cross-correlation scores between reconstructed and widefield reference images are shown as heatmaps for the different studied scenarios. **a** Sample simulated objects with different sparsity levels: 2^{12} (left) and 2^8 (right) bright points. **b** Results for the incoherent imaging case under different SNR conditions: SNR=5 (left), SNR=10 (middle), and SNR=∞ (right). **c** Coherent imaging results under the same SNR conditions: SNR=5 (left), SNR=10 (middle), and SNR=∞ (right). The horizontal axis is the number of realizations (M), and the vertical axis is the object sparsity. The color scale indicates the correlation coefficient from 0 to 1.

These results demonstrate that sparser objects can be reconstructed with fewer realizations, while complex objects need substantially more measurements. Higher SNR conditions predictably improve reconstruction quality, and coherent imaging appears to require an increased number of measurements compared to incoherent imaging for an equivalent fidelity.

7 Matrix-based scattering compensation algorithms

In this section, we provide the background and mathematical foundation for the I-CLASS algorithm used to reconstruct the target objects in our work. The I-CLASS algorithm builds upon the fundamental principles of reflection-matrix-based imaging techniques [1]. To understand its operation, we first establish the mathematical foundation of the reflection-matrix formalism for imaging through static scattering media, and explain the basics of the original CLASS algorithm, applied to imaging with deterministic controlled illuminations, such as plane waves or scanning beams [7, 8]. We then proceed to show how the matricial formalism and the CLASS algorithm were extended to the case of random illuminations via CTR-CLASS [1], and the extension to phase and amplitude correction by I-CLASS [2]. Finally, we explain how these principles apply to dynamic scattering by the exchange of roles between the scattering medium and the target object.

Reflection-matrix formalism

We begin by considering the simplest scenario of coherent imaging a reflective planar target object through a scattering layer. For simplicity, we assume isoplanatism, i.e., that the imaged field is a convolution of the field at the object plane by a complex-valued field point spread function (PSF). This is indeed the case when imaging objects that are smaller than the isoplanatic patch size[2, 9]. Under these conditions, the measured output field is given by:

$$E_{\text{out}}(\mathbf{r}) = P_{\text{det}}(\mathbf{r}) * E_{\text{obj}}(\mathbf{r}) \tag{2}$$

where $E_{\text{obj}}(\mathbf{r})$ is the reflected field at the object plane, and $P_{\text{det}}(\mathbf{r})$ represent the detection PSF, which is the result of the combined effect of the scattering medium and the imaging system, and $*$ denotes convolution.

The field reflected from the object at the object plane, $E_{\text{obj}}(\mathbf{r})$, is the product of the object reflectivity $O(\mathbf{r})$ and the illumination at the object plane. Since the considered scenario is of a target object that is hidden behind a thin scattering layer, the illumination at the target object plane is given by a convolution of the 'input' illumination field without the scattering medium present,$E_{\text{in}}(\mathbf{r})$, and the effective 'illumination PSF', $P_{\text{ill}}(\mathbf{r})$, that results from the scattering medium and potentially also the illumination system. Thus, the measured output field behind the scattering layer when an input illumination $E_{\text{in}}(\mathbf{r})$ is used is given by:

$$E_{\text{out}}(\mathbf{r}) = P_{\text{det}}(\mathbf{r}) * [O(\mathbf{r}) \cdot (P_{\text{ill}}(\mathbf{r}) * E_{\text{in}}(\mathbf{r}))] \tag{3}$$

When sampling these fields and PSFs on a discrete grid with N pixels, and arranging the fields into column vectors with N entries each, the linear relationship given by Supp.Eq. 3 above can be expressed in matricial form as:

$$\mathbf{E}_{\text{out}} = \mathbf{P}_{\text{det}}\mathbf{O}\mathbf{P}_{\text{ill}}\mathbf{E}_{\text{in}} \equiv \mathbf{R}\mathbf{E}_{\text{in}} \tag{4}$$

Here, $\mathbf{P}_{\text{ill}}$ and $\mathbf{P}_{\text{det}}$ are $N \times N$ Toeplitz (convolution) matrices representing the illumination and detection PSFs (with the shifted PSFs as their columns), and $\mathbf{O}$ is a diagonal matrix with the object complex-valued reflectivity on its diagonal. The product of the illumination matrix, object matrix, and detection matrix describes the propagation of any input field through the scattering medium to the object and back from the object through the scattering medium to the detection system. This matrix product forms the reflection matrix of the complex medium and the target object, which has a characteristic Toeplitz-Diagonal-Toeplitz (TDT) structure:

$$\mathbf{R} \equiv \mathbf{P}_{\text{det}}\mathbf{O}\mathbf{P}_{\text{ill}} \tag{5}$$

For a static scattering medium and object scenario, one can measure the reflection matrix, $\mathbf{R}$, column by column by illuminating the medium with $m = 1..N$ controlled input fields (basis vectors), and recording the output scattered light fields for each of these $m = 1..N$ modes, which in matricial form translates to:

$$\mathbf{E}_{\text{out},m} = \mathbf{R}\mathbf{E}_{\text{in},m} \tag{6}$$

Once the reflection matrix is measured, the challenge of reconstructing the image of the hidden object from the scattered light measurements is equivalent to decomposing the reflection matrix into its three components. The CLASS algorithm [10] is fundamentally a matrix decomposition method that can take any matrix with this TDT structure and decompose it into its constituent matrices. This allows recovery of the aberration-free object reflectivity function, $\mathbf{O}$, by separating it from the distortions introduced by the PSFs. Importantly, such a decomposition is made possible due to the fact that while the full reflection matrix contains $N \times N = N^2$ elements (measurements), it is the product of three matrices, where in each matrix there are only N unknown elements. Thus, the challenge is to retrieve the $3N$ unknowns (target object and two PSFs) from N^2 measurements.

The CLASS scattering-compensation algorithm

The CLASS algorithm [7, 11] decomposes a reflection matrix given by: $\mathbf{R} = \mathbf{P}_{\text{det}}\mathbf{O}\mathbf{P}_{\text{ill}}$ that possess a Toeplitz–Diagonal–Toeplitz (TDT) product structure, to the three matrices that compose it. Thus, simultaneously recovering, in an iterative manner, the object and the illumination and detection isoplanatic distortions.

The process begins by Fourier transforming the reflection matrix from real space to the Fourier space coordinates. Since in real space the reflection matrix has a Toeplitz–Diagonal–Toeplitz (TDT) product, after a two-dimensional Fourier transform, the reflection matrix in Fourier space coordinates possess a Diagonal–Toeplitz–Diagonal (DTD) structure:

$$\tilde{\mathbf{R}} = \tilde{\mathbf{P}}_{\text{det}}\,\tilde{\mathbf{O}}\,\tilde{\mathbf{P}}_{\text{ill}} \tag{7}$$

The diagonal structure of the illumination and detection isoplanatic distortions matrices in Fourier coordinates is easily understood as the complex amplitude transfer function (or the OTF in incoherent imaging) of the scattering medium, i.e. the

phase of a thin phase-mask scattering layer model. The CLASS algorithm operates under the assumption that both $\tilde{\mathbf{P}}_{\text{ill}}$ and $\tilde{\mathbf{P}}_{\text{det}}$ represent pure phase-only masks, i.e. $\tilde{\mathbf{P}}_{\text{ill}} = \text{diag}(e^{i\phi_1}, e^{i\phi_2}, \dots)$, where ϕ_k denotes the illumination phase distortion at the k-th Fourier component, and similarly for $\tilde{\mathbf{P}}_{\text{det}}$ with the detection-path phase distortions. We note that the assumption of phase-only distortions is relaxed in the recent I-CLASS algorithm [2], explained below.

To illustrate the basic working principle of CLASS, we present a simple 3×3 toy-model example that can be easily extended to the general $N \times N$ case:

1. Setting up the toy matrices. We define our matrices as:

$$\tilde{\mathbf{P}}_{\text{ill}} = \text{diag}\big(e^{i\phi_1}, e^{i\phi_2}, e^{i\phi_3}\big), \quad \tilde{\mathbf{O}} = \begin{bmatrix} \tilde{O}_1 & \tilde{O}_3 & \tilde{O}_2 \\ \tilde{O}_2 & \tilde{O}_1 & \tilde{O}_3 \\ \tilde{O}_3 & \tilde{O}_2 & \tilde{O}_1 \end{bmatrix},$$

and $\tilde{\mathbf{P}}_{\text{det}} = \text{diag}\big(e^{i\psi_1}, e^{i\psi_2}, e^{i\psi_3}\big)$.

2. Alternating correction approach. The key insight that is the basis for the CLASS algorithm is that the multiplication of $\tilde{\mathbf{O}}$ from the right by the diagonal matrix $\tilde{\mathbf{P}}_{\text{ill}}$ results in the multiplication of each k-th column of $\tilde{\mathbf{O}}$ by a single scalar value that is the phase distortion of the k-th component. In a similar fashion, the multiplication of $\tilde{\mathbf{O}}$ from the left by the diagonal matrix $\tilde{\mathbf{P}}_{\text{det}}$ simply multiplies each row of $\tilde{\mathbf{O}}$ by a different scalar value that represnets the detection distortions of this k-vector.

CLASS iteratively finds these scalar values (i.e. Fourier space phase distortions) by calculating the correlations between (e.g. neighboring) columns and rows. It is based on alternately correcting the illumination and detection distortions, where in each step it considers illumination-only distortions or detection-only distortions. Correcting one set of distortions, while temporarily ignoring the others.

For instance, to correct $\tilde{\mathbf{P}}_{\text{ill}}$, CLASS conceptually treat $\tilde{\mathbf{R}}$ as if it were simply $\tilde{\mathbf{O}}\tilde{\mathbf{P}}_{\text{ill}}$. In our toy-model example, this product is:

$$\tilde{\mathbf{O}}\tilde{\mathbf{P}}_{\text{ill}} = \begin{bmatrix} \tilde{O}_1 e^{i\phi_1} & \tilde{O}_3 e^{i\phi_2} & \tilde{O}_2 e^{i\phi_3} \\ \tilde{O}_2 e^{i\phi_1} & \tilde{O}_1 e^{i\phi_2} & \tilde{O}_3 e^{i\phi_3} \\ \tilde{O}_3 e^{i\phi_1} & \tilde{O}_2 e^{i\phi_2} & \tilde{O}_1 e^{i\phi_3} \end{bmatrix} \tag{8}$$

3. Column-shifting operation. A critical step in CLASS is to recognize that, due to the Toeplitz structure of $\tilde{\mathbf{O}}$, one can shift the columns of the matrix to align the object's Fourier-components. Specifically, we shift each column so that each row contains the same object component multiplied by a different phase factor:

$$(\tilde{\mathbf{O}}\tilde{\mathbf{P}}_{\text{ill}})_{\text{shifted}} = \begin{bmatrix} \tilde{O}_1 e^{i\phi_1} & \tilde{O}_1 e^{i\phi_2} & \tilde{O}_1 e^{i\phi_3} \\ \tilde{O}_2 e^{i\phi_1} & \tilde{O}_2 e^{i\phi_2} & \tilde{O}_2 e^{i\phi_3} \\ \tilde{O}_3 e^{i\phi_1} & \tilde{O}_3 e^{i\phi_2} & \tilde{O}_3 e^{i\phi_3} \end{bmatrix} \tag{9}$$

This alignment is the key that allows CLASS to extract the phase distortions by cross-correlating the different matrix columns.

4. Extracting phase information. To extract the phase distortions, CLASS performs two operations:

a) Calculate the mean of each row to obtain a vector $\mathbf{T}$:

$$\mathbf{T} = \begin{bmatrix} \tilde{O}_1 \cdot \frac{1}{3}(e^{i\phi_1} + e^{i\phi_2} + e^{i\phi_3}) \\ \tilde{O}_2 \cdot \frac{1}{3}(e^{i\phi_1} + e^{i\phi_2} + e^{i\phi_3}) \\ \tilde{O}_3 \cdot \frac{1}{3}(e^{i\phi_1} + e^{i\phi_2} + e^{i\phi_3}) \end{bmatrix} = \begin{bmatrix} \tilde{O}_1 M \\ \tilde{O}_2 M \\ \tilde{O}_3 M \end{bmatrix} \tag{10}$$

where $M = \frac{1}{3}(e^{i\phi_1} + e^{i\phi_2} + e^{i\phi_3})$ represents a mean phase term.

b) Calculate the scalar product of each of the matrix columns with the vector $\mathbf{T}$ to find their relative phase shift. This is performed in a matricial notation by multiplying the transpose of the shifted matrix by the conjugate of $\mathbf{T}$:

$$\mathbf{v} = (\tilde{\mathbf{O}}\tilde{\mathbf{P}}_{\text{ill}})^T_{\text{shifted}}\mathbf{T}^* = S \cdot |M| \cdot e^{-i\theta_M} \begin{bmatrix} e^{i\phi_1} \\ e^{i\phi_2} \\ e^{i\phi_3} \end{bmatrix} \tag{11}$$

where $S = |\tilde{O}_1|^2 + |\tilde{O}_2|^2 + |\tilde{O}_3|^2 > 0$ is a positive real scalar and θ_M is the phase of M.

The phase angle of each element in $\mathbf{v}$ now gives us:

$$\arg(\mathbf{v}) = \begin{bmatrix} \phi_1 - \theta_M \\ \phi_2 - \theta_M \\ \phi_3 - \theta_M \end{bmatrix} \tag{12}$$

This provides the relative phases of $\tilde{\mathbf{P}}_{\text{ill}}$ up to a global phase offset θ_M, which is physically irrelevant as only relative phases matter for image reconstruction.

5. Applying correction for the illumination distortions. In this step, each matrix column is multiplied by the conjugate of the phase distortion, to 'align' all columns. This is performed in matricial form by creating a correction matrix using the phase distortions estimated in the previous step:

$$\tilde{\mathbf{P}}_{\text{ill}}^{\text{correction}} = \text{diag}(e^{-i\arg(v_1)}, e^{-i\arg(v_2)}, e^{-i\arg(v_3)}) \tag{13}$$

And applying this correction to the reflection matrix columns:

$$\tilde{\mathbf{R}}_{\text{corrected}} = \tilde{\mathbf{R}} \cdot \tilde{\mathbf{P}}_{\text{ill}}^{\text{correction}} \tag{14}$$

6. Detection phase distortions correction. After correcting the illumination distortion, CLASS performs the detection distortion by taking the transpose of the corrected reflection matrix, and repeating the same steps (3-5) to correct the detection distortions, as the rows and columns are now switched. $\tilde{\mathbf{P}}^T_{\text{det}}$ now plays the role previously held by $\tilde{\mathbf{P}}_{\text{ill}}$, allowing us to apply the same procedure to correct the detection distortion.

7. Iterative refinement. The illumination and detection correction steps are alternated iteratively until the phase estimates converge. Convergence of this process under single-scattering conditions is proven in [7], and usually requires a few tens of iterations.

8. Reconstruction and extraction. After convergence, applying the final phase correction to all matrix columns (and rows) results in an aberration-free reflection matrix, where each column represents an aberration-free frame. One can then use any single frame as the reconstruction or coherently compound the different frames to reconstruct an improved image, which is equivalent to reconstructing a confocal image from plane wave illumination.

CTR-CLASS and I-CLASS: retrieving a reflection matrix from measurements using unknown input fields

In the case that the input fields for measuring the reflection matrix cannot be controlled or are unknown, one can still measure the resulting scattered light field and retrieve a 'virtual reflection matrix' from the covariance matrix of these fields. This concept for 'compressive time-reversed' (CTR) measurement of the reflection matrix was introduced by Lee et al. [1], and forms the basis for what is termed a CTR-CLASS approach. In CTR-CLASS, instead of acquiring the full matrix by N measurements, one illuminate the object with $M \leq N$ different random and unknown fields, $S_m(\mathbf{r}) = P_{\text{ill}} * E_{\text{in},m}(\mathbf{r})$. For each illumination, the field equation becomes:

$$E_{\text{out},m}(\mathbf{r}) = P_{\text{det}}(\mathbf{r}) * [O(\mathbf{r})S_m(\mathbf{r})] = P_{\text{det}}(\mathbf{r}) * O_m(\mathbf{r}) \tag{15}$$

where $O_m(\mathbf{r}) = O(\mathbf{r})S_m(\mathbf{r})$ represents the m-th reflected field from the object.

Arranging these M measurements as columns of a 'measurement matrix', $\mathbf{A}$, we have:

$$\mathbf{A} = \mathbf{P}_{\text{det}}\mathbf{O}\mathbf{S} \tag{16}$$

Where $\mathbf{S}$ is a matrix that contains the illumination patterns at the object plane,$S_m(\mathbf{r})$, as its columns.

Importantly, if the illumination patterns are random and uncorrelated, the covariance matrix of $\mathbf{A}$ takes the form:

$$\mathbf{A}\mathbf{A}^\dagger = \mathbf{P}_{\text{det}}\mathbf{O}(\mathbf{S}\mathbf{S}^\dagger)\mathbf{O}^\dagger\mathbf{P}_{\text{det}}^\dagger \approx \mathbf{P}_{\text{det}}|O|^2\mathbf{P}_{\text{det}}^\dagger \tag{17}$$

where we have used the fact that $\mathbf{S}\mathbf{S}^\dagger \approx \mathbf{I}$ for uncorrelated illuminations.

The result of Supp.Eq. 17 shows that the covariance matrix of scattered light fields obtained under random unknown illumination has the same TDT structure as the conventional reflection matrix (Supp. Eq. 5) with $|O|^2$ replacing O. Thus, the CLASS algorithm can be applied directly to this covariance matrix to retrieve both the absolute value of the object reflectivity from $|O|^2$, and the detection PSF, P_{det}.

While the CLASS algorithm was developed to compensate for phase-only distortions, i.e. to the case where the illumination or detection PSF are each given by a Fourier transform of a phase-only mask, the recently introduced I-CLASS algorithm [2] extends the CLASS correction (or equivalently, matrix decomposition) to the case where both amplitude and phase distortions exists.

Dynamic scattering correction: exchange of roles between the target object and the scattering PSF

In the dynamic scattering scenario considered in this work, the fundamental imaging equation (Eq. 15) changes. Instead of considering the static scattering medium (PSF) and $m = 1..M$ varying illuminations of the conventional reflection matrix acquisition, which can be described by:

$$E_{\text{out},m}(\mathbf{r}) = P_{\text{det}}(\mathbf{r}) * O_m(\mathbf{r}) \tag{18}$$

where $O_m(\mathbf{r})$ is the field reflected from the object at the m-th illlumination, we consider a static object imaged by a time-varying PSF that results from the dynamics of the scattering medium. The imaging equation in this case becomes:

$$E_{\text{out},m}(\mathbf{r}) = P_m(\mathbf{r}) * O(\mathbf{r}) = O(\mathbf{r}) * P_m(\mathbf{r}) \tag{19}$$

Where P_m is the PSF for the m-th realization of the dynamic scatterer. The crucial insight presented in our work is that, due to the commutativity of the convolution operation, the equation for the dynamic medium case has exactly the same mathematical form as the random illumination case of conventional reflection matrix acquisition, but with the roles of the object and PSF interchanged.

Due to this mathematical equivalence of the imaging equation between the dynamic scattering and random illumination scenarios (Supplementary Eqs. 15 and 19), the covariance matrix of the acquired dataset 'measurement matrix' $\mathbf{A}$ has the same TDT structure as that of the covariance matrix of CTR-CLASS, and thus the I-CLASS algorithm can be used to reconstruct the object by naively applying it on the covariance matrix.

We note that to achieve a fixed object illumination throughout the different measurements in the coherent imaging case, we utilized an illumination spot size at the scattering layer plane that is smaller than the correlation length of the diffuser (see Supplementary Section 5 on "Effect of illumination spot size").

I-CLASS algorithm

The I-CLASS algorithm [2] extends the CLASS algorithm [10] estimation of phase distortions to allow both phase and amplitude distortions in conventional reflection matrix measurements. Specifically, in I-CLASS the phase corrections are found in the exact same manner as the CLASS algorithm. Following the standard CLASS phase-correction, I-CLASS estimates the amplitude distortions in the Fourier domain (the Modulation Transfer Function, MTF) from the diagonal of the covariance matrix in the Fourier domain. This amplitude information is used to correct for the amplitude aberrations by Fourier-reweighting.

In the conventional case of a static medium using multiple illuminations [2], the I-CLASS algorithm produces an estimate of the Fourier phase and amplitude of the scattering PSF. However, in the case of a dynamic scattering medium considered in this work, applying the I-CLASS algorithm (without any change to the data or the algorithm) produces the Fourier phase and amplitude of the target object. All that is

left is to Fourier transform (or digitally propagate) the algorithm output to reconstruct the image of the object at the target plane.

8 Captions for movies

Video S1.

Measured experimental uncorrected frames of the target object, as presented in Fig. 2b-e of the main text, displayed alongside their estimated PSFs and the I-CLASS reconstruction. Scale bars, 100 µm.

Video S2.

Measured experimental uncorrected frames of the target object, as presented in Fig. 2f-i of the main text, displayed alongside their estimated PSFs and the I-CLASS reconstruction. Scale bars, 100 µm.

References

[1] Lee, H., Yoon, S., Loohuis, P., Hong, J.H., Kang, S., Choi, W.: High-throughput volumetric adaptive optical imaging using compressed time-reversal matrix. Light: Science & Applications **11**(1), 16 (2022)

[2] Weinberg, G., Sunray, E., Katz, O.: Noninvasive megapixel fluorescence microscopy through scattering layers by a virtual incoherent reflection matrix. Science Advances **10**(47), 5218 (2024)

[3] Bertolotti, J., Van Putten, E.G., Blum, C., Lagendijk, A., Vos, W.L., Mosk, A.P.: Non-invasive imaging through opaque scattering layers. Nature **491**(7423), 232–234 (2012)

[4] Katz, O., Heidmann, P., Fink, M., Gigan, S.: Non-invasive single-shot imaging through scattering layers and around corners via speckle correlations. Nature photonics **8**(10), 784–790 (2014)

[5] Labeyrie, A.: Attainment of diffraction limited resolution in large telescopes by fourier analysing speckle patterns in star images. Astronomy and Astrophysics, Vol. 6, p. 85 (1970) **6**, 85 (1970)

[6] Fienup, J.R.: Phase retrieval algorithms: a comparison. Applied optics **21**(15), 2758–2769 (1982)

[7] Kang, S., Kang, P., Jeong, S., Kwon, Y., Yang, T.D., Hong, J.H., Kim, M., Song, K.-D., Park, J.H., Lee, J.H., *et al.*: High-resolution adaptive optical imaging within thick scattering media using closed-loop accumulation of single scattering. Nature communications **8**(1), 2157 (2017)

[8] Yoon, S., Lee, H., Hong, J.H., Lim, Y.-S., Choi, W.: Laser scanning reflection-matrix microscopy for aberration-free imaging through intact mouse skull. Nature communications **11**(1), 5721 (2020)

[9] Bertolotti, J., Katz, O.: Imaging in complex media. Nature Physics **18**(9), 1008–1017 (2022)

[10] Choi, W., Kang, M., Hong, J.H., Katz, O., Lee, B., Kim, G.H., Choi, Y., Choi, W.: Flexible-type ultrathin holographic endoscope for microscopic imaging of unstained biological tissues. Nature communications **13**(1), 4469 (2022)

[11] Kang, S., Yoon, S., Choi, W.: Implementation of reflection matrix microscopy: An algorithm perspective. Journal of Physics: Photonics **7**(2), 023002 (2025)
