# Supplementary material for: Matrix-based imaging through dynamic scattering
Source: Nat Commun. 2025 Oct 24;16:9413. doi: 10.1038/s41467-025-64422-x (PMC12552628; doi:10.1038/s41467-025-64422-x)
Supplement: Supplementary file 2 — Description of additional supplementary files [file 41467_2025_64422_MOESM2_ESM.pdf]

Description of Additional Supplementary Files

Supplementary Movie 1: Measured experimental uncorrected frames of the target object, as presented in Fig.2b-e of the main text, displayed alongside their estimated PSFs and the I-CLASS reconstruction.

Supplementary Movie S2: Measured experimental uncorrected frames of the target object, as presented in Fig.2f-i of the main text, displayed alongside their estimated PSFs and the I-CLASS reconstruction.
